# Semantic biclustering for finding local, interpretable and predictive expression patterns

**DOI:** 10.1186/s12864-017-4132-5

**Published:** 2017-10-16

**Authors:** Jiří Kléma, František Malinka, Filip železný

**Affiliations:** Department of Computer Science, Czech Technical University in Prague, Karlovo náměstí 13, 121 35 Prague 2, Czech Republic

**Keywords:** Biclustering, Enrichment analysis, Symbolic machine learning, Ontology, Gene expression

## Abstract

**Background:**

One of the major challenges in the analysis of gene expression data is to identify local patterns composed of genes showing coherent expression across subsets of experimental conditions. Such patterns may provide an understanding of underlying biological processes related to these conditions. This understanding can further be improved by providing concise characterizations of the genes and situations delimiting the pattern.

**Results:**

We propose a method called semantic biclustering with the aim to detect interpretable rectangular patterns in binary data matrices. As usual in biclustering, we seek homogeneous submatrices, however, we also require that the included elements can be jointly described in terms of semantic annotations pertaining to both rows (genes) and columns (samples). To find such interpretable biclusters, we explore two strategies. The first endows an existing biclustering algorithm with the semantic ingredients. The other is based on rule and tree learning known from machine learning.

**Conclusions:**

The two alternatives are tested in experiments with two Drosophila melanogaster gene expression datasets. Both strategies are shown to detect sets of compact biclusters with semantic descriptions that also remain largely valid for unseen (testing) data. This desirable generalization aspect is more emphasized in the strategy stemming from conventional biclustering although this is traded off by the complexity of the descriptions (number of ontology terms employed), which, on the other hand, is lower for the alternative strategy.

## Background

The general goal of *biclustering* (or *block-clustering, co-clustering*) [1] is to find interesting submatrices in a given data matrix. A submatrix is defined by a subset of rows and a subset of columns of the original matrix. In other words, it is a compact rectangular section of a matrix that can be obtained by permuting the rows and columns (respectively) of the input matrix. There are multiple ways to define the interestingness of biclusters; the simple view adopted here is that the biclusters cover as many as possible 1’s within the containing binary matrix while leaving out as many as possible 0’s. Biclustering has become remarkably popular in bioinformatics [2], especially in gene expression data analysis tasks [3, 4]. Here, biclustering detects an expression specific to a subset of genes in a subset of samples (situations).

*Semantic clustering* denotes conventional clustering augmented by the additional requirement that the discovered clusters are characterized through concepts defined as prior domain knowledge. The characterizations are obviously requested for the sake of easy interpretation of the analysis results. A popular activity in bioinformatics, where (ordinary) clusters of genes with similar expressions profiles are first detected and *enrichment analysis* [5] is subsequently applied on such clusters, is in fact an example of (‘manual’) semantic clustering. The two steps in the latter workflow can also be merged into a single phase as demonstrated in [6, 7]. Semantic clustering is also related to the subgroup discovery approach [8], although in an unsupervised setting. The term semantic clustering is also employed in the software-engineering context [9] and captures a roughly similar meaning as in the present context.

In this study we explore the combination of the two concepts, that is *semantic biclustering*. Specifically, we want to be able to detect biclusters as outlined above; however, we also want their elements to share a joint description as in semantic clustering. In the case of biclustering, the description pertains to both the rows (that is, genes) as well as the columns (that is, situations). We follow this goal because formal ontologies are frequently available and relevant to either dimension of the input data matrix. An example of such a data set is the *Dresden ovary table* [10, 11]. Simply put, our goal is to design an algorithm able to detect biclusters characterized e.g. as “glucose metabolism genes in late developmental stages” whenever such genes in such stages are uniformly expressed. To the best of our knowledge, the previous approaches most related to semantic biclustering are [12], where formal knowledge associated with both rows and columns of a data matrix is used to specify filters for detected patterns and [13, 14], which aim at biclustering of gene expression data with biclusters coherent in terms of gene functional annotation. The authors of [15] proposed a new iterative bi-clustering algorithm and applied it to a binary gene set expression dataset, i.e., the dataset where expression of whole gene sets was captured. They worked with the semantic annotation of the original gene expression data, but they employed the semantics solely in the preprocessing step.

In the rest of the paper we formalize the problem of semantic biclustering first. Then, we propose two strategies for semantic biclustering and test them comparatively on two experimental datasets. Our contributions also include a design of a suitable validation protocol, as evaluation criteria are not fully evident in unsupervised data analysis.

## Methods

### Problem formalization

We assume a set of genes *Γ*, a set of situations *Σ*, and a binary set of expression indicators {0,1}. We further assume a joint probability distribution over these three sets *p*:{0,1}×*Γ*×*Σ*→[0;1]. In a gene-expression assay, a set *G*⊆*Γ* of genes and set *S*⊆*Σ* of situations are selected and expression is sampled for all pairs of the selected genes and situations. In other words, a matrix $\mathbbm {A}=(a_{g,s})$, *g*∈*G*, *s*∈*S* is formed such that *a*_*g*,*s*_=1 with *p*(1|*g*,*s*) (0 otherwise).

In standard multivariate analysis of gene expression, $\mathbbm {A}=(a_{g,s})$ represents a *sample set* in the sense that a *sample* corresponds to a column in $\mathbbm {A}$. For benefits of statistical inference, it is typically assumed that samples are independent and identically distributed (i.i.d.); more precisely, that *S* is drawn i.i.d. from the marginal *p*(*s*). Note that the drawing is with replacement, so strictly speaking *S* (and *G* analogically) is a multi-set rather than a set. This distinction is however immaterial in the present context. In the present biclustering context, we put genes and situations (rows and columns) on equal footing. That is to say, a sample corresponds to a single measurement *a*_*g*,*s*_. Under this view, the sample set {(*a*_*g*,*s*_,*g*,*s*):*g*∈*G*,*s*∈*S*} is not an i.i.d. sample from *p*(*a*,*g*,*s*) even if both *G* and *S* are i.i.d. samples from the respective marginals *p*(*g*) and *p*(*s*), which is due to the sample set’s rectangularity. Indeed, if the latter contains a sample for a particular pair (*g*,*s*), it will necessarily also contain all pairs (*g* ^′^,*s*),*g* ^′^∈*G* and all pairs (*g*,*s* ^′^),*s* ^′^∈*S*, so the samples are mutually dependent.

#### Ordinary biclusters

A *bicluster* in matrix ${\mathbbm {A}}=(a_{g,s}), g \in G, s \in S$ is a submatrix defined by a subset of rows and columns, i.e., a tuple (*G* ^′^,*S* ^′^) where *G* ^′^⊆*G* and *S* ^′^⊆*S*. A *system of biclusters* of $\mathbbm {A}$ is *B*={(*G*_*k*_,*S*_*k*_)} where (*G*_*k*_,*S*_*k*_) are biclusters in ${\mathbbm {A}}$. The *extension of B* is

1 $$ ext(B) = \{(g,s) : g \in G', s \in S', (G',S') \in B\}  $$

A usual requirement is that a system of biclusters covers regions of ${\mathbbm {A}}$ that are *homogeneous* regarding the contained values. This may be interpreted in multiple ways and here we adhere to the simplest interpretation that the bicluster system *B* should ideally include all 1’s present in ${\mathbbm {A}}$ and exclude all 0’s. Then a natural quality measure of *B* counts 1’s inside its extension and 0’s outside of it

2 $$ \sum_{(g,s) \in ext(B)} a_{g,s} + \sum_{(g,s) \in G \times S \setminus ext(B)} 1-a_{g,s}  $$

For convenience, we introduce an *indicator function* *b*:*G*×*S*→{0,1}

3 $$ b(g,s) = 1\ \text{iff}\ (g,s) \in ext(B)  $$

which allows us to rephrase the above quality measure as |{(*g*,*s*)∈*G*×*S*:*a*_*g*,*s*_=*b*(*g*,*s*)}|. Normalizing this to the interval [0;1], one obtains the formula

$$\widehat{Acc}(b) = \frac{|(g,s) \in G\times S: a_{g,s} = b(g,s)\}|}{|G||S|} $$

which is known as the *training (in-sample) accuracy* of *b* viewed as a classifier. This quantity provides an empirical approximation to the true *b*’s accuracy on *G*×*S*, which is *p*(*g*,*s*,*b*(*g*,*s*)|(*g*,*s*)∈*G*×*S*) according to our probabilistic model. The conditional part is important since *b*’s domain is restrained to *G*×*S*. On one hand, this classification viewpoint provides an additional motivation to maximize the ad-hoc formula (2). On the other hand, viewing $\widehat {Acc}$ as a proxy for the true accuracy entails certain problems.

First, as we have commented already, the sample set where $\widehat {Acc}$ is determined is not i.i.d. as normally required for a training set, although this could be tolerated if the intended use of $\widehat {Acc}$ is as a heuristic guiding the search for *B*, rather than as an unbiased estimator. Second, $\widehat {Acc}$ can be trivially maximized by a system of single-element biclusters covering exactly all 1’s in $\mathbbm {A}$. Such an *overfitting* solution is commonplace in classification and is usually avoided by an additional *regularization* term. Here, the latter could penalize small biclusters, or alternatively a high number of them. So one would search *B* maximizing

$$\widehat{Acc}(b) + \lambda/|B| $$

with *λ* determining the trade-off between accuracy and the size of the bicluster system. In fact, a regularizer is normally added to formula 2 in biclustering algorithms [16, 17] to prevent the trivial solution, irrespectively of any classification context.

The third problem lies in the restriction of *b* onto the *G*×*S* domain, which does not enable us to use *b* on genes and situations not in the training set. At first sight, this does not seem a problem if one is not interested in using the bicluster system *B* for classification. However, it makes the assessment of *B*’s quality problematic in the following sense. Besides the training accuracy $\widehat {Acc}$ acting as a search heuristic, we are also interested in an unbiased estimate of the quality of the final system *B* produced by the biclustering algorithm. An ideal quality measure is the true accuracy *p*(*g*,*s*,*b*(*g*,*s*)) of *b*, which would normally be estimated using a *hold-out* or *testing* data set *T* *e* *s* *t*={(*g*_*k*_,*s*_*k*_,*a*_*k*_)} drawn i.i.d. from *p*(*g*,*s*,*a*), as

4 $$ Acc(b) = \frac{|\{(g_{k},s_{k}, a_{k}) \in Test: a_{k} = b(g_{k},s_{k})\}|}{|Test|}  $$

However, this value cannot be established as *b* is not defined for arguments with values outside the training sample set and—to our best intelligence—there is no sensible way in which the bicluster system *B* could induce a classifier beyond the *G*×*S* domain. We will see in turn that this problem is overcome elegantly by *semantic biclusters*.

#### Semantic biclusters

Here we consider biclusters which are not defined by an enumeration of the selected rows and columns, but rather by enumerating conditions according to which the rows and columns are selected. In particular, the conditions are represented by semantic annotation terms pertaining to genes (rows) and situations (columns). Formally, we assume a set of gene annotation terms *γ*, and analogically situation annotation terms *σ*. Furthermore, relations *R*_*γ*_⊆*G*×*γ*, *R*_*σ*_⊆*S*×*σ* are defined, associating genes and situations with selected annotation terms.

For an arbitrary gene set *G*, a term set *T* ^*γ*^⊆*γ* induces the set {*g*∈*G*:∀*t*∈*T* ^*γ*^,(*g*,*t*)∈*R*_*γ*_} of exactly those genes in *G* that comply with all the terms in *T* ^*γ*^. We denote this induced set as *G*(*T* ^*γ*^). Similarly for a situation set *S* and a situation term set *T* ^*σ*^, *S*(*T* ^*σ*^)={*s*∈*S*:∀*t*∈*T* ^*s*^,(*s*,*t*)∈*R*_*σ*_}.

Thus within a matrix of genes *G* and situations *S*, a *semantic bicluster* (*T* ^*γ*^,*T* ^*σ*^) induces a unique ordinary bicluster (*G*(*T* ^*γ*^),*S*(*T* ^*σ*^)), and a *system of semantic biclusters* $SB = \left \{\left (T^{\gamma }_{k}, T^{\sigma }_{k}\right)\right \}$ defines a unique ordinary system of biclusters *B*. Due to this correspondence between *SB* and *B*, *SB* can be searched using the heuristic $\widehat {Acc}(B)$ we elaborated above.

Unlike the extension of an ordinary system of biclusters (Eq. 1), the extension *e* *x* *t*(*S* *B*) of a system of semantic biclusters *SB* is not confined to the matrix of genes *G* and situations *S*

5 $${} ext(SB) = \{(g,s) : g \in \Gamma(T^{\gamma}), s \in \Sigma(T^{\sigma}), (T^{\gamma}, T^{\sigma}) \in SB\}  $$

and thus also the indicator function *s* *b*:*Γ*×*Σ*→{0,1} defined as in (3) now has all genes and situations in its domain. (Note that the restriction of *e* *x* *t*(*S* *B*) to the matrix *G*×*S* coincides with the extension *e* *x* *t*(*B*) of the ordinary system *B* of biclusters defined by *SB*; this is easy to see by replacing *Γ* and *Σ* respectively by *G* and *S* in Eq. 5).

This means that for a system *SB* of semantic biclusters, we can obtain an extra-sample (testing) quality estimate *A* *c* *c*(*s* *b*) per Eq. 4 which was not possible with ordinary biclusters. Note that the testing sample set *T* *e* *s* *t*={(*g*_*k*_,*s*_*k*_,*a*_*k*_)} needed for the estimate is drawn i.i.d. from *p*(*g*,*s*,*a*) and is not expected to form a matrix. This has a positive practical implication for the evaluation procedure, which will be commented further in the experimental section.

#### Soft semantic biclusters

The last extension we introduce is that of *soft* semantic biclusters, motivated by the fact that in the terms sets *T* ^*γ*^, *T* ^*σ*^ defining a semantic bicluster (*T* ^*γ*^, *T* ^*σ*^), some of the terms may be more important than others. The reason for this will follow from the algorithm implementations elaborated below. Here we simply assume that the sets *T* ^*γ*^, *T* ^*σ*^ consist of pairs (*t*,*w*) where *t*∈*γ* (*t*∈*σ*) and the weight *w*∈(0;1]. In this situation, we adapt the classification function to

6 $$ \begin{aligned} sb(g,s) = 1 &\textrm{ iff} \qquad (T^{\gamma},T^{\sigma}) \in SB \\ &\text{and} \sum_{(t,w) \in T^{\gamma}, (g,t) \in {R_{\gamma}}} w \geq \theta_{G} \\ &\text{and} \sum_{(t,w) \in T^{\sigma}, (g,t) \in {R_{\sigma}}} w \geq \theta_{S} \\ \end{aligned}  $$

where *θ*_*G*_,*θ*_*S*_∈*R* are some real thresholds (hyper-parameters). Informally, the classifiers outputs 1 iff at least one of the biclusters in *SB* *supports* the classified tuple (*g*,*s*). The tuple is supported by a bicluster (*T* ^*γ*^,*T* ^*σ*^) if the weights of terms which are simultaneously (i) assumed by *T* ^*γ*^ (*T* ^*σ*^, respectively), (ii) and among the annotations of *g* (*s*), sum up to at least *θ*_*G*_ (*θ*_*S*_). The earlier definitions of $\widehat {Acc}$ and *A* *c* *c* apply to this redefined classifier *sb* as well.

### Algorithms

At least two different strategies lend themselves to find a good system of semantic biclusters *SB*. The first option is to find a system *B* of ordinary biclusters first, and then identify the characteristic annotation terms *T* ^*γ*^ and *T* ^*σ*^ for each of the biclusters in *B*. The second option is to search directly in the space of (sets of) semantic biclusters, i.e. explore systematically various combinations of the annotation terms. We explore both strategies henceforth. In the first one we employ an existing biclustering algorithm and subject its results to an *enrichment analysis* [5] algorithm, revealing annotation terms which are enriched on either dimension of the produced biclusters. The alternative strategy is materialized by an arrangement of classical symbolic machine-learning techniques known as decision rule and tree learning [18]. It is implemented in terms of two closely related methods that share the preprocessing step and differ in the consecutive learning step.

#### Bicluster enrichment analysis

The enrichment approach to semantic biclustering first searches for a set of ordinary biclusters. The goal is to find a small set of biclusters that cover as many 1’s as possible and as few 0’s as possible. In other words, we search for the most concise biset-based description that minimizes the occurrence of false positives and false negatives. In the field of biclustering, this is a well-known task that can be tackled with approximate pattern matching [17, 19, 20], non-negative matrix decomposition [21, 22], bipartite graph partitioning [23] or heuristic algorithms [24–27]. The bicluster semantics are disregarded for the moment.

In our approach, we employed the popular PANDA+ tool [17] to accomplish the first step. PANDA+ adopts a greedy search that iteratively builds a sequence of biclusters. The constructed bicluster set gradually increases its coverage of the input matrix. This bicluster set is initially required to be noise-less, i.e. without false positives. In a subsequent step, PANDA+ extends the biclusters by allowing false positives. The main guiding parameter is the level of accepted noise which may be used to balance between the size of the description (the number of biclusters and their size) and the quality of the description (the amount of false predictions). $\mathbbm {A}$ has to be transformed into the FIMI sparse format [28] before calling PANDA+.


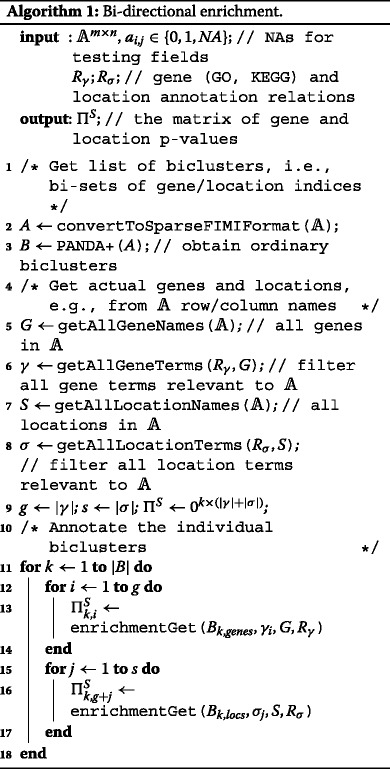


In the second step, the biclusters are annotated in terms of prior domain knowledge, i.e., their semantics are revealed. In our case, we use the gene ontology (GO) terms [29, 30] and KEGG terms [31] to annotate the individual genes. The dedicated Drosophila location ontology (DLO) terms [10] and Drosophila anatomy ontology (DAO) terms [32] were used to annotate the situations; in particular, these terms define the developmental stages and anatomical locations of the sample. Each non-trivial bicluster (comprising more than 1 gene and 1 stage) is annotated by all the terms (GO+KEGG and situation/anatomy ontology, respectively) whose enrichment exceeds the predefined statistical significance threshold. In order to avoid this hyperparameter in our workflow, we propose setting the threshold automatically within the permutation-based test that compares the bicluster enrichment scores with the scores reached in permuted gene expression matrix. The significance threshold is set to guarantee that the false discovery rate for annotation terms in real biclusters remains small. The individual terms are scored proportionally to their statistical significance, yielding the weights *w* assumed by the classification principle in Eq. 6. We employed the topGO Bioconductor package [33] to find the GO terms and the Fisher test to reveal the KEGG and location ontology terms enriched in the individual biclusters.

This approach to semantic biclustering could as well be referred to as *bi-directional enrichment*. The procedure pseudocode is shown in Algorithm 1. Despite the NP-complexity of the general problem of finding the optimal set of biclusters [2], the suboptimal heuristic algorithm is computationally scalable. The size of the input matrix influences mainly the initial bicluster search; time complexity of PANDA+ is $\mathcal {O}(|B|mn^{2})$ [17] where |*B*| is the number of biclusters and *m*=|*G*|,*n*=|*S*| are the dimensions of the expression matrix. The sizes |*γ*|, |*σ*| of the annotation vocabularies influence solely the annotation step whose time complexity is $\mathcal {O}(|B|(|{\gamma }|*m+|{\sigma }|*n))$.

#### Rule and tree learning

The alternative approach is based on a reduction of the problem to a classification-learning problem. This entails a transformation of the original data matrix $\mathbbm {A}$ into an auxiliary binary matrix $\mathbbm {M}$ of dimensions (|*G*|·|*S*|)×(|*γ*|+|*σ*|+1). Matrix $\mathbbm {A}$ is unrolled into $\mathbbm {M}$ so that each row of $\mathbbm {M}$ corresponds to one element *a*_*i*,*j*_ of $\mathbbm {A}$ and has the form

7 $$ t_{1}, t_{2}, \ldots t_{|{\gamma}|}, t_{|{\gamma}|+1}, t_{|{\gamma}|+2}, \ldots t_{|{\gamma}|+|{\sigma}|}, \textit{expression}  $$

where the first |*γ*| numbers are binary indicators of annotation terms (acquiring a value of 1 iff the corresponding term is associated with gene in *i*’th row of $\mathbbm {A}$), the subsequent |*σ*| numbers are analogical indicators of situation ontology-terms for situation in *j*’th column of $\mathbbm {A}$, and the last number is the expression indicator for the said gene and situation, and thus equals *a*_*i*,*j*_. The transformation details are shown in Algorithm 2.


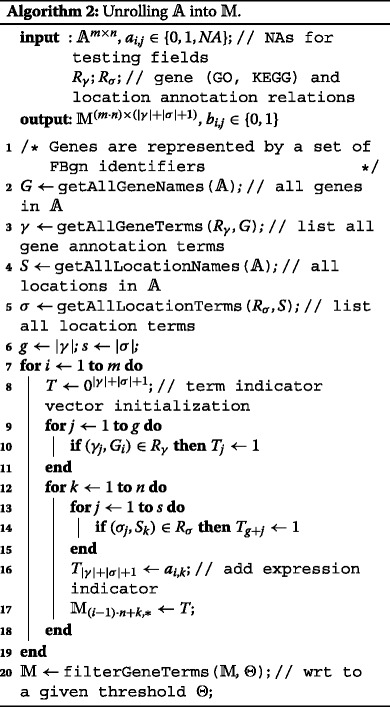


The next step is learning a classification model to predict *expression* from *t*_1_,…*t*_|*γ*|+|*σ*|_. To this end, $\mathbbm {M}$ represents the training data with individual rows such as (7) corresponding to learning examples with the last element being the class indicator. The model we search for takes the form of a list of conjunctive decision rules [18], each of which acquires the form

8 $$ \wedge_{i \in I} t_{i} \wedge_{j \in J} t_{j+|\gamma|} \rightarrow \textit{expression}  $$

where the rule conditions *I* ⊆ [1;|*γ*|], *J* ⊆ [1;|*σ*|] are learned selections of gene and situation ontology terms. The rule stipulates that a gene annotated with all the gene-ontology terms indexed by *I* is likely to be expressed in situations annotated with all the situation-ontology terms indexed by *J*. If no rule in the learned rule set predicts expression for a pair (*g*,*s*), the rule set defaults to the no-expression prediction.

Consider the set *P*=*G*×*S* containing all the gene-situation pairs (*g*,*s*) satisfying the conditions of rule (8). It is easy to see that *P* forms a submatrix of $\mathbbm {A}$, i.e., there exists a permutation of $\mathbbm {A}$’s rows and columns making *P* a rectangular section of $\mathbbm {A}$. Indeed, *G* identifies a set of rows and *S* identifies a set of columns. The conjunction in (8) is satisfied perfectly by the genes in the intersection of *G* and *S*, which is thus a rectangle. Therefore, each rule such as (8) identifies a bicluster in $\mathbbm {A}$. Note that the rectangular property essentially follows from the propositional-logic form of the rule and would not hold true for the more general *relational* rules considered in [8].

Moreover, a rule set optimized for classification accuracy on training data such as (7) will produce those biclusters of $\mathbbm {A}$ which contain a high number of 1’s. Indeed, perfect training-set accuracy is achieved if and only if the biclusters represented by the rules in the rule-set collectively cover all the 1’s and no 0’s in $\mathbbm {A}$.

Summarizing the two observations, the learned rule set represents a set of biclusters of $\mathbbm {A}$, each of which is homogeneous in that it collects positive indicators of expression. Furthermore, each such bicluster is characterized by the ontology terms *G* and situation terms *S* found in the corresponding rule such as (8). Thus, the procedure described does indeed convey the semantic biclustering task.

In addition, we propose a variation to the workflow described, in which the rule-set learner is replaced by a *decision tree* learner [18]. Each vertex in a learned tree corresponds to one ontology term, and the test represented by the vertex determines whether the term is among the annotation of the classified pair of gene and situation. Since all the attributes (including the class attribute) of the training data (7) are binary, the learned tree is also binary. Each path from the root to one positive leaf can be rewritten as a rule in the form (8), except that some of the literals may be negated. For example, literal ¬*t*_1_ expresses the condition that *t*_1_ is *not* among the annotation terms. So the learned decision tree defines a set of semantic biclusters as the rule-set does, except these biclusters are defined in a more expressive language (allowing negation) than we considered in the original formalized model.

The main reason for exploring this decision tree alternative is that it is often claimed that decision trees exhibit performance superior to that of decision rule sets.

In our implementation of this approach, we used the JRip and J48 algorithms from the WEKA machine-learning software [34] to learn the rule-sets and decision trees, respectively. The JRip algorithm is an implementation of a propositional rule learner, Repeated Incremental Pruning to Produce Error Reduction (RIPPER) [35]. J48 is an implementation of the well-known C4.5 algorithm [36].

The time complexity of this approach is determined by the complexity of converting the $\mathbbm {A}$ into $\mathbbm {M}$, which is $\mathcal {O}(mn(|\gamma |+|\sigma |))$, and the complexity of the subsequent learning algorithm. In the case of binary decision trees, the runtime of the heuristic J48 algorithm grows linearly with the number of training instances and quadratically with the number of features [37], in our problem it is $\mathcal {O}(mn(|\gamma |+|\sigma |)^{2})$. As the total number of annotation terms can be large, the actual runtime of this approach would be much larger than for the bi-directional enrichment. For this reason, we perform a feature selection step prior to the learning step. The published JRip’s time complexity [35] implies the learning complexity for our problems $\mathcal {O}(mn\text {log}^{2}(mn))$. In other words, a large number of samples in $\mathbb {M}$ indicates a time consuming run if compared to the other methods implemented in our work.

### Evaluation procedure

Both biclustering and enrichment analyses are unsupervised data mining methods and the exact way of validating their performance is not obvious. For example, perfectly homogeneous biclusters can usually be found at the cost of their very small size. The size and homogeneity should thus be traded-off but their relative importance would have to be set apriori. Similarly, the semantic annotations discovered may either represent genuine characteristics of the biclusters, or the included terms may be enriched merely by chance. Distinguishing these two effects through a statistical test involves distributional assumptions which we cannot guarantee.

We solve the latter dilemma by measuring the quality of semantic biclusters from the point of view of *predictive classification*, and particularly using an extra-sample (testing) accuracy estimate as proposed in Eq. 4. This assumes that the available data is split randomly into a training partition where the semantic biclusters are found, and a testing partition where they are evaluated. The training split is a (strict) submatrix of the input matrix and thus its complement (the testing split) is not a matrix. Fortunately, a matrix form is not required of the testing split as explained in the Problem formalization section.

As stated already, the strategy based on conventional biclustering and subsequent enrichment analysis results in a set of soft semantic biclusters inducing the classification principle described by Eq. 6. The latter depends on the two hyper-parametric thresholds *θ*_*G*_ and *θ*_*S*_, and their different choices result in different values of the accuracy measure (4). In such a situation, it is convenient to visualize the global performance profile through *ROC analysis*. Here, the accuracy measure (4) is decomposed into the *false positive rate* component *FPr* and the *true positive rate* *TPr*, both of which are functions of *θ*_*G*_ and *θ*_*S*_. By varying these hyperparameters, a set of (*F* *P* *r*,*T* *P* *r*) points is obtained, forming the *ROC curve*. The area under this curve (termed AUC) represents the quality of the classifier for the entire range of the hyperparameters. The semantic biclustering validation procedure is summarized in Algorithm 3.

The approach based on rule and tree learning produces crisp semantic biclusters, and as such it induces classifiers in the standard form given by (3). For the sake of unified comparison, we also evaluate these classifiers through ROC analysis although they do not contain explicit threshold parameters. This is made possible by the employed JRip and J48 algorithms which provide confidence values along with the expression predictions. We make a positive expression call only if the corresponding confidence value exceeds a threshold *Θ*, and we obtain the ROC curve by varying *Θ*.


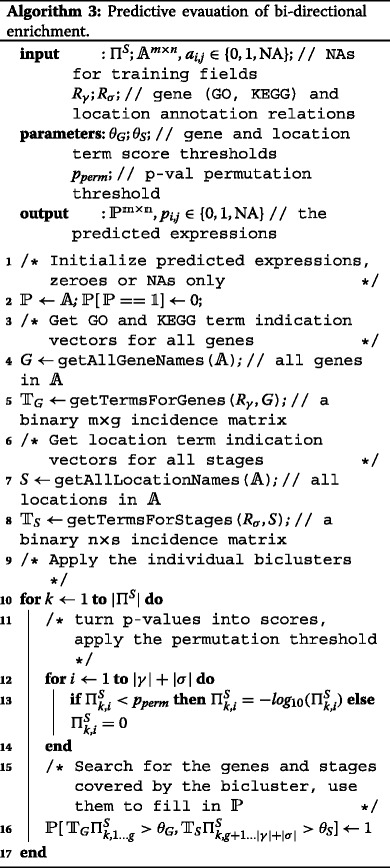


## Results

### Experimental datasets

We conducted our experiments on two real datasets. The first one is the Dresden ovary table [10]. The table captures the distribution of different mRNA molecules in various cell types involved in oocyte production in the ovary of female Drosophila melanogaster flies. The authors of the table believe [11] that the resource can be used to gain insight into specific genetic features that control the distribution of mRNAs and this insight may be instrumental in cracking the ‘RNA localization code’ and understanding how it affects the activity of proteins in cells. In this problem, the dedicated situation ontology (available from the same source) describes Drosophila ovary segments and their developmental stages. The ontology is in fact a location term hierarchy that binds the locations available in the Dresden ovary table by the relations part_of and develops_from. As such, the hierarchy deals with 100 terms. The gene ontology was used in its standard available form [29, 33] including 8,407 GO terms in total. The set of KEGG terms was considerably smaller, we dealt with 133 terms that annotated a limited set of 1605 genes. For this reason, the importance of KEGG is smaller than that of GO. After minor data cleansing, the expression matrix has 6510 rows (genes) and 100 columns (situations) with 47.5% positive data instances. The detailed data statistics can be found in Table 1.

**Table 1:** Drosophila ovary table statistic

	Complete dataset	Train	Test		
		all	keepLocations	keepGenes	bd
#of rows/genes	6,510	5,447	1,063	5,447	1,063
#of columns/locations	100	84	84	16	16

**Fig. 1:**
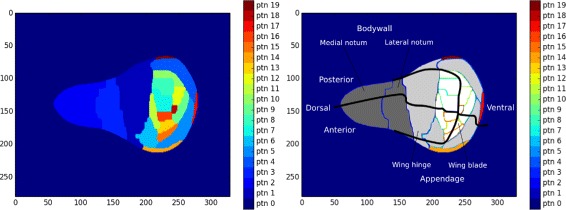
Segmentation of an imaginal disc. An example of segmentation of an imaginal disc (left), altogether with its annotation by the Drosophila ontology terms (right). The disc is split into 20 segments distinguished in colors, the split was found to best capture the gene expression patterns observed in the individual in situ hybridization images. The annotation stems from [40]

The second experimental dataset comes from the same organism, i.e., Drosophila melanogaster, and captures the spatial gene expression in the larval imaginal discs (IDiscs). An imaginal disc is a part of insect larva from which the adult body parts develop. The dataset is a binary representation of an automatically processed large collection of fluorescent in situ 2D hybridization images. The images were collected for more than 1000 genes in 4 different imaginal discs (wing, antenna-eye, leg and haltere). About 20 distinct locations (image segments) were distinguished for each disc, see Fig. 1 for further details. A set of semantically annotated biclusters may help to reveal and understand the local expression patterns in larval development. Altogether, the binary imaginal disc dataset contains the expression of 1207 genes in 72 different locations with 75.4% positive data entries. The detailed data statistics can be found in Table 2. Similarly to the Dresden ovary table, we assigned a set of GO and KEGG terms to each gene. 114 KEGG terms appeared in the annotation records of 423 distinct genes. Further, each segment of a particular imaginal disc was manually assigned a set of DAO terms. The DAO consists of over 8000 terms with broad coverage of Drosophila anatomy including the descriptions of imaginal discs and their compartments, we made use of 148 distinct terms. The summary ontology term counts are available in Table 3.

**Table 2:** Imaginal disc dataset statistic

	Complete dataset	Train	Test		
		all	keepLocations	keepGenes	bd
#of rows/genes	1,207	1,010	197	1,010	197
#of columns/locations	72	60	60	12	12

**Table 3:** The number of annotation terms available for our experimental datasets

	GO	KEGG	DAO	DLO
Ovary	8,407	1,605	-	100
IDisc	5,083	423	147	-

For the evaluation purposes, each data set was randomly split into a submatrix containing 70% of the original matrix elements, and the complement which was used as the testing set.

### Experimental protocol

The bicluster enrichment method was run with the PANDA+ noise parameters that minimized the total cost of biclusters in the training set (i.e., the summarizing criterion that controls both bicluster size and the number of false positives and negatives). This setting can be reached in a fully unsupervised way and avoids both too noisy and too detailed sets of biclusters. For the ovary dataset, the statistical significance thresholds were set to 0.05 for genes and 0.1 for situations. For the imaginal disc dataset, the statistical significance thresholds were set to 0.01 for genes and 0.1 for situations. The reason for different values between the gene dimension and the situation dimension is that the number of situations is lower than the number of genes and the location ontology is less complex than the gene annotation. For this reason, even less significant location terms prove helpful when generalizing to unseen data. The method was run repeatedly with the following sets of match thresholds: *θ*_*G*_∈{1,5,10,50} and *θ*_*S*_∈{1,5,10,50}. The results in ovary dataset suggested that precision decreases slowly with decreasing match thresholds while recall grows quite rapidly. The best precision/recall trade-off is thus achieved for the minimum match threshold values *θ*_*G*_=*θ*_*S*_=1. The size of bicluster description does not directly change with the match threshold values, their decrease raises the number of genes and developmental stages matched by bicluster annotation terms. To the contrary, in imaginal discs we were able to find biclusters with strongly related location terms. For this reason, *θ*_*S*_=50 seems to be the best threshold as it already provides a sufficient recall and its decrease only leads to decreasing precision.

The rule and tree learning was performed with the default WEKA parameters for JRip and J48. In order to work with a reasonable number of features, feature selection was employed first. All the features (annotation terms) of the train matrix (originating from the $\mathbbm {M}$ matrix) that occurred in fewer than approximately 1‰ expression entries (the train matrix rows) were removed. The cut-off threshold was found with the feature frequency histograms. Eventually, we worked with a train matrix size of 457,548 ×326 and 60,600 ×403, respectively. Besides speeding up the learning process, we avoided the annotation terms that cannot generalize over a reasonable number of locations.

Table 4 shows the results including the AUROC achieved by the two proposed strategies (the rule and tree learning strategy is represented by the rule learning method and the tree learning method, they are evaluated independently) as well as further information regarding the found biclusters. The table summarizes 10 experimental runs, each for a different random train-test split. Note that the traditional cross-validation scenario cannot be applied in the two-dimensional setting. AUROC evaluates the proposed methods from the point of view of their generalization ability. Importantly, both the proposed strategies generalize far better than random. In other words, the semantic descriptions of the biclusters can be used to predict the expression for combinations of genes and situations not present in training data.

**Table 4:** Evaluation results of the proposed approaches to semantic biclustering

Dataset	Method	AUROC	# of biclusters	# of terms per bicluster
Ovary	Bicluster Enrichment	0.823 ±0.006	11.8 ±1.5	64.8 ±3.4
	Rules (JRip)	0.636 ±0.01	102.6 ±21.5	7.1 ±0.61
	Tree (J48)	0.659 ±0.01	109.9 ±5.2	25.4 ±2.0
IDiscs	Bicluster Enrichment	0.608 ±0.03	16.4 ±4.7	47.9 ±2.13
	Rules (JRip)	0.565 ±0.01	25.9 ±6.2	7.89 ±0.53
	Tree (J48)	0.627 ±0.05	20.6 ±11.09	11.01 ±4.71

## Discussion

The bicluster enrichment method seems to be the most reliable predictive method in datasets that can be described by a coherent biclusters whose size allows their reliable subsequent annotation. In the ovary dataset, the mean bicluster size exceeded 30,000 entries and the biclusters proved to generalize well. If given an unseen pair of positive (present) and negative (absent) expression entries, it correctly guesses the positive entry with more than a 82% chance. On the other hand, the method employs a large number of bicluster annotation terms to reach a reasonable recall. In our experiments, the average number of GO, KEGG and location terms per bicluster was 59, 2 and 4 respectively (as the KEGG and location ontology deal with a smaller number of terms). This number of terms may make the interpretation hard for a human expert. At the same time, in more fragmented and difficult domains such as the imaginal disc dataset, the mean size of biclusters drops (we observed the mean bicluster size 3,998 in the imaginal disc dataset) and the biclusters seem to generalize worse. J48 proved to be the method that copes well with this increased fragmentation. The decision tree grows without an immediate decrease in its generalization power. JRip outputs the most concise bicluster description, its disadvantages lie in the low AUROC and by far the slowest runtime.

The experimental results conform to expectations. The bicluster enrichment method ignores the semantic description when building the biclusters. Consequently, they tend to faithfully fit the expression matrix and compactly represent the expression patterns that the matrix contains. On the other hand, their postponed semantic annotation may turn out complex and fuzzy. The rule and tree learning does just the opposite; it directly searches for concise semantic descriptions that separate positive and negative expression values in training data. As a result, the descriptions have a tendency to be short and crisp with potentially lower recall. Table 5 evaluates biological homogeneity of the found biclusters in terms of their enrichment. The table shows the proportion of generated biclusters that have at least one enriched annotation term in each dimension at the level of significance 0.05. As the rule and tree learning methods directly define biclusters by the annotation terms, their proportions are naturally high. Biclusters without an annotation in one of the directions may originate namely if a bicluster is defined solely by one type of terms (either gene, or location terms). The proportions of enriched biclusters reached by bi-directional enrichment are lower but satisfactory too. We ascribe it to the PANDA’s ability to cope with noise and search for large and semantically interpretable biclusters. The biological homogeneity is comparable with the result published in [14], where homogeneity in gene dimension only was measured.

**Table 5:** Biological homogeneity of the found biclusters in terms of their enrichment

Dataset	Method	% enriched
Ovary	Bicluster Enrichment	0.952 ±0.063
	Rules (JRip)	0.981 ±0.017
	Tree (J48)	0.974 ±0.021
IDiscs	Bicluster Enrichment	0.851 ±0.102
	Rules (JRip)	0.962 ±0.041
	Tree (J48)	0.931 ±0.043

**Fig. 2:**
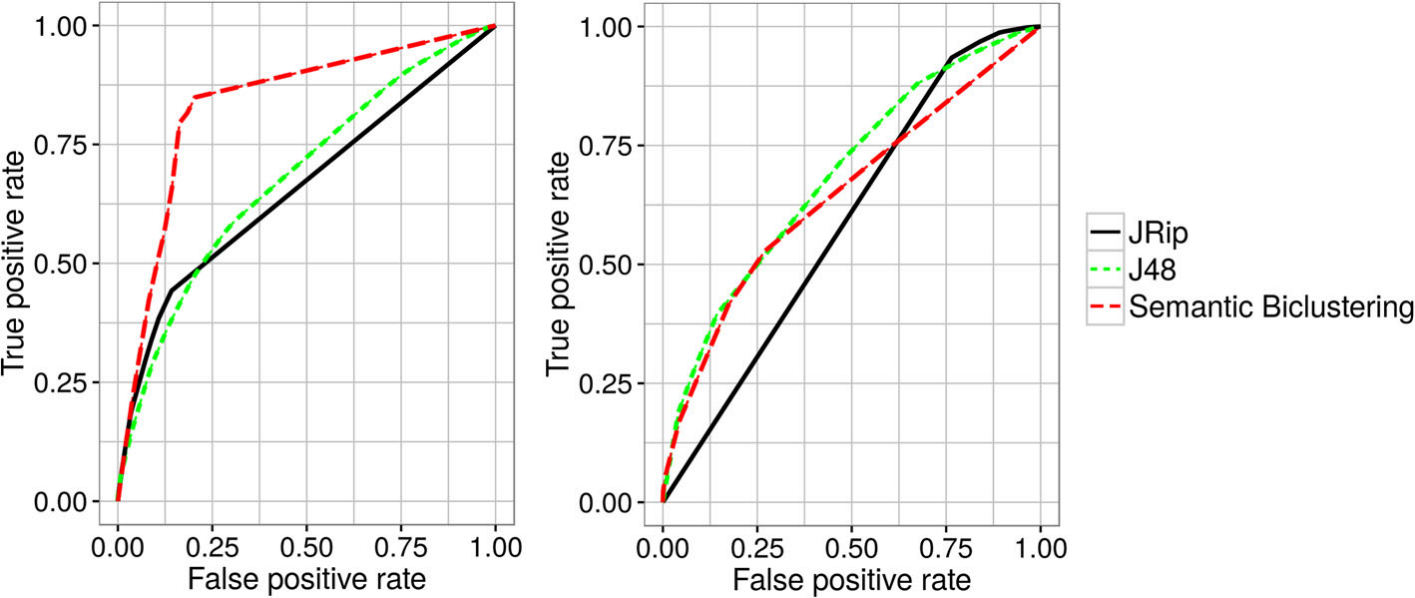
Semantic biclustering ROC curves for Drosophila ovary table (left) and Imaginal disc dataset (right)

Figure 2 presents the individual ROC curves. For the bicluster enrichment method, the curve is constructed as a convex hull for 16 binary classifiers reached for different *θ*_*G*_ and *θ*_*S*_ settings. However, the curve suggests that one of the classifiers (namely the one for *θ*_*G*_=*θ*_*S*_=1) makes the major contribution to the aggregate AUROC while the other classifiers approach the trivial convex hull or fall under it. J48 and JRip can provide both binary and probabilistic outcomes. Here, we work with the probabilistic outcome, the curve is constructed with different probability thresholds for assigning an example to the positive class.

**Fig. 3:**
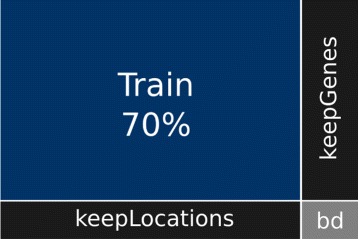
Train and test matrices

Eventually, we compared the generalization ability independently in terms of gene and location annotation terms. Under this evaluation protocol, the test matrices were split into three parts, see Fig. 3. The first submatrix denoted as *kG* (keepGenes), contains only the rows whose gene identifiers were already observed in the complementary train set while its columns correspond to the locations that were not observed there. Consequently, each biclustering method has to generalize towards the locations. The second submatrix denoted as *kL* (keepLocations), covers the locations already observed in the train set and the previously unobserved genes. Each biclustering method has to employ gene annotation terms to be able to predict here. Finally, the third submatrix *bd* contains the rest of testing entries. Bi-directional generalization has to be applied here. The results are summarized in Table 6. The main conclusion is that it is much easier to generalize in terms of locations than in terms of genes. The locations common for a bicluster tend to share location annotation terms observed for other genes with a similar local expression pattern. On the contrary, the description in terms of genes is often extensive with more difficult application to external genes. The bicluster enrichment method provides the best generalization for the *bd* region, where both the genes and locations were previously unseen.

**Table 6:** Generalization in terms of genes and locations. The table compares the AUROC for three different settings

Dataset	Method	kG	kL	bd
Ovary	Bicluster Enrichment	0.929 ±0.013	0.677 ±0.03	0.818 ±0.014
	Rules (JRip)	0.692 ±0.02	0.583 ±0.01	0.583 ±0.02
	Tree (J48)	0.725 ±0.002	0.604 ±0.01	0.604 ±0.02
IDiscs	Bicluster Enrichment	0.705 ±0.06	0.560 ±0.02	0.593 ±0.03
	Rules (JRip)	0.588 ±0.01	0.546 ±0.01	0.537 ±0.02
	Tree (J48)	0.630 ±0.06	0.627 ±0.05	0.602 ±0.04

*kG* tests the generalization across locations, *kL* the generalization across genes and *bd* the generalization in both the dimensions

Runtimes of all the three implemented methods are summarized in Tables 7 and 8. All tests were performed with the same configuration: 8-core Intel Xeon E5-2630v3 2.40 GHz. We measured runtimes in 10 experimental runs with different random train-test splits. The tables distinguish the individual subtasks that underlie the implemented methods. Table 8 for bi-directional enrichment distinguishes the preparatory subtask (data and ontology upload, train-test split preparation), the model building (biclustering in PANDA+) and the model testing (annotation of the individual biclusters and their application to test data). Table 7 splits the runtime between the ARFF building (process of unrolling the gene expression matrix into the ARFF file), the model building (learning of decision trees or rule sets) and the model testing (the application of the trees or rules to test data). The runtimes show that biclustering enrichment method is in the order of magnitude faster than rule and tree learning. Firstly, it is the result of large semantic description as discussed during the theoretical complexity analysis. Secondly, it stems from efficient implementation of PANDA+ in C while the rest of the code runs in R, Perl and Java. Consequently, only the building of ARFF file in rule and tree learning takes more time than bi-directional enrichment. These two reasons also contribute to the fact that bicluster annotation and application to test data is more time consuming than bicluster construction in bi-directional enrichment. It is also clear that JRip algorithm is much less computationally efficient than J48.

**Table 7:** Runtimes (in seconds) of rule and tree learning methods on DOT and IDiscs datasets. The process of transforming original matrix onto ARFF file (build ARFF) and the process of building classification models were measured separately

Split	DOT				IDiscs					
	Build ARFF	Build model		Test model		Build ARFF	Build model		Best model	
		J48	JRip	J48	JRip		J48	JRip	J48	JRip
1	1,033	1,237	26,810	17.00	23.44	274	59.59	510.84	3.08	3.11
2	1,091	1,503	21,384	19.45	18.67	272	38.03	557.92	2.93	3.19
3	1,042	1,076	19,519	19.09	18.19	287	71.62	363.00	3.16	3.16
4	1,096	1,300	20,054	17.59	19.07	270	64.65	438.87	3.16	3.25
5	1,127	2,010	20,605	18.61	21.22	278	39.47	941.30	3.20	3.64
6	1,121	1,999	24,568	19.38	18.69	260	39.77	550.50	3.11	3.05
7	1,097	1,656	25,279	18.90	18.60	281	47.61	288.14	2.98	3.00
8	1,058	1,087	22,459	26.47	18.48	269	44.00	641.16	3.14	3.26
9	1,023	1,236	14,062	17.81	18.24	288	54.83	201.10	3.25	2.91
10	1,268	1,583	27,299	18.81	21.07	276	42.83	506.14	2.96	3.06
$\bar {x}$	1,096	1,469	22,204	19.31	19.57	629.4	50.24	499.9	3.10	3.16
*s* *d*(*x*)	±70.6	±343	±3,995	±2.64	±1.75	±32.3	±11.78	±204.8	±0.11	±0.2

**Table 8:** Runtimes (in seconds) of bi-directional enrichment on DOT and IDiscs datasets

Split	DOT			IDiscs		
	Prepare data	Build model	Test model	Prepare data	Build model	Test model
1	21.80	74.75	278.79	14.75	133.14	70.42
2	20.44	122.27	233.85	13.96	112.36	53.41
3	14.76	100.80	259.17	10.11	101.49	49.12
4	16.05	87.42	223.64	9.36	107.10	47.32
5	14.54	120.49	266.52	9.28	72.78	60.17
6	16.98	110.70	228.80	13.87	124.81	45.06
7	14.79	100.55	231.63	9.51	153.33	82.83
8	14.43	80.02	229.41	14.08	144.09	50.18
9	14.58	94.29	204.34	9.73	176.95	61.83
10	14.02	103.77	230.10	15.60	90.13	45.86
$\bar {x}$	16.24	99.51	238.63	12.03	121.62	56.62
*s* *d*(*x*)	±2.73	±15.88	±22.46	±2.61	±31.26	±12.30

## Conclusion

We have motivated and defined the task of semantic biclustering and proposed two strategies to solve the task, based on adaptations of current biclustering, enrichment, and rule and tree learning methods. We compared them in experiments with Drosophila ovary and imaginal disc gene expression data. Our findings indicate that the bicluster enrichment method achieves the best performance in terms of the area under the ROC curve, at the price of employing a large number of ontology terms to describe the discovered biclusters.

In future work, the statistical implications of the non-standard way of splitting the data matrix into the (rectangular) training set and the testing set could be investigated. Furthermore, a method for semantic biclustering that would combine the complementary advantages of the proposed approaches could be devised. In principle, the biclustering enrichment ignores prior knowledge when searching for biclusters. None of the biclusters have to be interpretable as a result. The rule and tree-based methods directly stem from prior knowledge and search for the most general conjunctive concepts that fit the training data at the risk of their overfitting. Finally, a biological interpretation of the results reached in particular domains could be provided.

We made the project publicly available through GitHub [38]. The repository contains source code of both the implemented strategies as well as both the experimental datasets.
